# miR-215-5p regulates osteoporosis development and osteogenic differentiation by targeting XIAP

**DOI:** 10.1186/s12891-022-05731-w

**Published:** 2022-08-17

**Authors:** Zilong Yin, Jian Shen, Qiang Wang, Liangyuan Wen, Wenjing Qu, Yaonan Zhang

**Affiliations:** 1 Department of Orthopaedics, Beijing Hospital, National Center of Gerontology, Institute of Geriatric Medicine, Chinese Academy of Medical Sciences, Beijing, People’s Republic of China; 2 Department of Surgery, Tongzhou Maternal and Child Health Hospital of Beijing, 124 Yuqiao Middle Road, Tongzhou District, Beijing, 101100 People’s Republic of China

**Keywords:** miR-215-5p; XIAP, Osteoporosis, Osteogenic differentiatioz

## Abstract

**Background:**

Osteoporosis (OP) is a metabolic disease that involves microstructure destruction and fracture damage. The present study probed into the significance of miR-215-5p in OP progression.

**Methods:**

Serum samples were collected from surgical patients and healthy controls. qRT-PCR analysis was utilized to determine the miR-215-5p level in clinical samples and human bone mesenchymal stem cells (hBMSCs) induced by β-glycerol phosphate. A dual luciferase reporter assay was exploited to examine the targeted relationship between miR-215-5p and XIAP. The mineralization and calcium deposition of hBMSCs were assessed by detection of ALP activity, Alizarin red staining, and osteoblast marker expression. Protein expression was determined by western blot analysis.

**Results:**

MiR-215-5p was significantly reduced in patients with OP and increased in hBMSCs treated with β-glycerophosphate. Enhanced miR-215-5p level triggered augment in osteoblast markers (Alkaline phosphatase/ ALP, Osteocalcin/ OCN, and Runt-Related Transcription Factor 2/ Runx2), which was accompanied by the increase of ALP activity in hBMSCs and accumulation of Calcium. Functional experiments show that XIAP was a target of miR-215-5p and negatively modulated by miR-215-5p. XIAP expression levels were increased in OP samples, and decreased XIAP in β-glycerophosphate-treated hBMSCs inhibited its’ osteogenic differentiation. Functional loss and acquisition experiments depicted that miR-215-5p promoted the differentiation of hBMSCs by inhibiting the XIAP level, playing a protective role in the pathogenesis of OP.

**Conclusions:**

β-glycerophosphate promoted the osteogenic differentiation of hBMSCs, increased miR-215-5p level, and decreased XIAP. miR-215-5p stimulated osteogenic differentiation of hBMSCs by targeting XIAP, shedding new insights for the detection and therapy of OP.

**Supplementary Information:**

The online version contains supplementary material available at 10.1186/s12891-022-05731-w.

## Introduction

Osteoporosis (OP) is a common metabolic bone disease characterized by deterioration of bone microstructure and a decrease in bone mass [1]. The common method of screening for OP is bone mineral density (BMD). According to the International Society of Clinical Densitometry (ISCD), the OP is defined by a BMD < 80 mg/cm^3^ [2]. Since BMD decreases gradually with age, it makes the elderly a good candidate for OP. The prevalence of OP in the elderly population in China is as high as 32.0% [3].

Pittenger, a foreign scholar, isolated and extracted a class of cells with multidirectional differentiation potential from iliac bone marrow, and defined them as bone marrow mesenchymal stem cells (BMSCs) [4]. BMSCs are found in the bone marrow tissues of human and other animal organisms and are widely used in scientific research due to their abundant source and easy access to materials. Due to the natural inherent property of multidirectional differentiation of BMSCs, induced differentiation of BMSCs into osteoblasts can provide a stable cell source for bone growth, bone repair, and anti-osteoporosis [5, 6]. Hence, the ability of hBMSCs to differentiate into osteoblasts could measure the development of OP, as described before [7, 8].

MicroRNA (miRNA) is an endogenous family of small RNA with a length of about 18–25 nucleotides [9]. miRNAs specifically bind to non-coding regions of mRNAs to inhibit the translation of mRNA target proteins, regulating the related cellular functions, such as cell proliferation, differentiation, autophagy, apoptosis, and other physiological and pathological processes [10, 11]. miRNAs also play significant roles in the field of bone regeneration [12], particularly in disrupting the dynamic equilibrium of bone reconstruction and bone formation during surgical evolution [13, 14]. It has been shown that a variety of miRNAs are involved in the osteogenic differentiation process of hBMSCs. For example, miR-19b-3p, miR-218, and miR-128 have been shown to promote osteogenic differentiation of BMSCs, thereby increasing bone mass and enhancing bone quality [15–17]. In addition, miR-23a, miR-532-3p, miR-3658 have been shown to inhibit osteogenic differentiation of hBMSCs, decrease bone mass and increase the risk of bone fragility [18–20]. Studies have revealed that, miR-215-5p is remarkably decreased in the serum samples of osteoporosis patients, which could be potential diagnostic biomarkers for osteoporosis [21]. However, the regulatory mechanism of miR-215-5p in OP pathogenesis remained obscure.

Here, we collected OP serum specimens and evaluated the clinical impact of miR-215-5p in OP diagnosis. Moreover, osteogenic differentiation of hBMSCs was constructed by treating hBMSCs with β-glycerophosphate and L-ascorbic acid. The underlying mechanism of the miR-215-5p/XIAP axis in mediating osteogenic differentiation was verified by observing mineralization and calcium deposits in hBMSCs.

## Materials and methods

### Specimen collection

Fifty-two patients who underwent OP treatment and 43 healthy control subjects who underwent routine physical examination from January 2018 to July 2020 at Beijing Hospital were recruited for our study. After 8 h fasting, serum sample was collected from all subjects and stored at -80 °C until analysis. Exclusion criteria: (1) use of estrogens, glucocorticoids, androgens, anabolic steroids within six months before surgery; (2) use of thiazide diuretics within two months before surgery; (3) history of surgery, trauma, tumor or tuberculosis. Each participant provided informed consent and the study obtained permission from the Ethics Committee of Beijing Hospital, National Center of Gerontology; Institute of Geriatric Medicine, Chinese Academy of Medical Sciences (2022BJYYEC-129–01).

### Cell culture and osteogenic differentiation induction

hBMSCs were obtained from American Culture Collection Company (ATCC, USA) and cultured in DMEM-F12 (Gibco, USA) with 10% fetal bovine serum (Takara, Japan) and 1% penicillin–streptomycin. Cells were cultured at 37 °C in an incubator containing 5% CO_2_. To stimulate osteogenic differentiation, hBMSCs were cultured in an osteogenic differentiation medium containing 10 mM β-glycerophosphate (Sigma-Aldrich, USA) and 50 ng/ml L-ascorbic acid (Sigma-Aldrich, USA). The medium is renewed every 3 days.

### Cell transfection

miR-215-5p mimics, miR-215-5p inhibitor and negative controls (miR-NC) were purchased from Takara (Japan). At the same time, Genepharma supplied si-XIAP and its negative control si-NC. All these oligonucleotides were transfected into hBMSCs using Lipofectamine RNAIMAX transfection reagent (Thermo Fisher Scientific, Inc., Shanghai, China) in accordance with the instructions provided with the appliance.

#### Quantitative reverse transcription polymerase chain reaction (qRT-PCR) analysis

Total RNA was extracted from serum and cells using a Trizol reagent (ElabScience, USA), and the purity of total RNA was verified by the Nanodrop method. The RNA was then reverse transcribed into cDNA using the SYBR-qPCR kit (Sigma-Aldrich, MO, USA) using 400 ng of RNA as a template. Reverse transcribed cDNA samples were subjected to quantitative fluorescent PCR reaction using SYBR qRT-PCR Master Mix (Thermo Fisher Scientific, Inc., Shanghai, China) on an ABI 7500 PCR instrument (Applied Biosystems, Beijing, China). Thermal cycling program: 95 °C for 1 min, 95 °C for 15 s, 60 °C for 15 s, 72 °C for 45 s, total 35 cycles. Amplification results were analyzed by the 2^−△△Ct^ method. U6 and GAPDH acted as internal controls. The primer sequence was as follows: miR-215-5p (Forward), 5’-ATGACCTATGAATTG-3’, and miR-215-5p (Reverse), 5’-GTGCAGGGTCCGAGGT-3’; WNK1 (Forward), 5’-ACCTAGTGTACCTGCAGTGGTG-3’, and WNK1 (Reverse), 5’-TTGCTGAGACACCTGGGAAG-3’; RB1 (Forward), 5’-CACAACCCAGCAGTTCAATATC-3’, and RB1 (Reverse), 5’-TGAGATCACCAGATCATCTTCC-3’; CTCF (Forward), 5’-ACCAACCAGCCCAAACAGAAC-3’, and CTCF (Reverse), 5’-GTATTCTGGTCTTCAACCTGAATGATAG-3’; RUNX1 (Forward), 5’-AACCCTCAGCCTCAGAGTCA-3’, and RUNX1 (Reverse), 5’-CAATGGATCCCAGGTATTGG-3’; XIAP (Forward), 5’- GGCGCCTACAAGAGGAGAAG- 3’, and XIAP (Reverse), 5’- GGCGCCTACAAGAGGAGAAG- 3’; U6 (Forward), 5’-ATTGGAACGATACAGAGAAGATT-3’, and U6 (Reverse), 5’-GGAACGCTTCACGAATTTG-3’; GAPDH (Forward), 5’-TGTGGTCATGAGTCCTTCCA-3’, and GAPDH (Reverse), 5’-ATGTTCGTCATGGGTGTGAA-3’.

### Western blot assay

The treated hBMSCs were collected to extract the total protein and BCA method was exploited to determine protein concentration. The quantitative protein was loaded by SDS-PAGE, transferred to a PVDF membrane, and blocked in 5% skim milk for 2 h at 37℃. Then add XIAP (1:1000, AB229050, Abcam, Shanghai, China), ALP (1:1000, AB229126, Abcam, Shanghai, China), OCN (1:1000, AB133612, Abcam, Shanghai, China), Runx2 (1:1000, AB236639, Abcam, Shanghai, China), and GAPDH (1:1000, AB9485, Abcam, Shanghai, China) antibodies and incubate overnight at 4 °C. Afterward, secondary antibodies (1: 1000, AB96899, Abcam, Shanghai, China) were added and incubated at room temperature for 1 h. After washing with TBST, the film was developed with an ECL luminescent solution. The development of the exhibition. The image was collected by the gel image analysis system, and the gray level of the strip was analyzed using ImageJ software.

### Measurement of ALP activity

ALP activity was determined using an ALP assay kit (ElabScience, USA). After cells were captured, they were lysed with a lysis buffer. The supernatant was recovered after centrifugation and transferred to a 96-well plate. The supernatant was then incubated with p-nitrophenyl phosphate (PNPP) and reaction buffer for 10 min at 37 °C. Finally, the absorbance value was determined at 570 nm by enzyme labeling.

### Alizarin red staining

hBMSCs treated during the logarithmic growth period were routinely digested with 1 ml of 0.2% trypsin–EDTA and centrifuged at 1800 rpm for 5 min at room temperature. Cell density was adjusted to 1.0 × 10^4^ cells/mL, inoculated into a 12-well plate, and cultured in a 5% CO_2_ incubator at 37 °C. After 21 days of incubation, the culture medium was removed from the pore plate using a sterile pipette. Rinse the plate gently twice with PBS solution to remove the culture medium from the surface. 2 ml of 4% neutral formaldehyde solution was added and fixed for 30 min. Then, aspirate the formaldehyde solution from the pore plate, gently clean it twice with the PBS solution and transfer it to a high magnification orthogonal optical microscope for observation.

### Dual-luciferase reporter assay

The StarBase (https://starbase.sysu.edu.cn/index.php) and TargetScan v7.2 (http://www.targetscan.org) was used to predict the potential interacting miRNAs. XIAP 3’-UTR (XIAP WT) with a miR-215-5p binding sequence was amplified by PCR and the reporter vector pmiRGlo (Promega, USA) was inserted. The mutation of the binding region in XIAP3’-UTR (XIAP-MUT) was successfully constructed using a fast-changing targeted mutation kit (Stratagene, USA). The constructed pmiRGlo-Xiap 3’-UTR vector was co-transfected into hBMSCs using a Lipofectamine RNAIMAX transfection reagent (Thermo Fisher Scientific, Inc., Shanghai, China). After 48 h of incubation, luciferase activity was determined using a luciferase dual reporting system (Promega, USA). Luciferase activity was measured with a photometer and normalized to Renilla luciferase activity.

### Statistical analysis

Experimental data were measured three times repeatedly and results were expressed by X ± standard deviation (SD). Data analysis was performed using SPSS 23.0 software, and GraphPad Prism 8.0.1 graphics software plotted the data. Student’s *t*-test was used to compare the two data sets, single factor analysis of variance was used to compare multiple data sets, and Pearson’s test was used to perform data correlation analysis. ROC curve analysis was performed as mentioned before to evaluate the diagnostic values of plasma miR-215-5p for osteoporosis [22]. Differences with *P* < 0.05 were statistically significant. The difference was statistically significant at *P* < 0.05.

## Results

### Clinical significance of miR-215-5p in OP

**Fig. 1:**
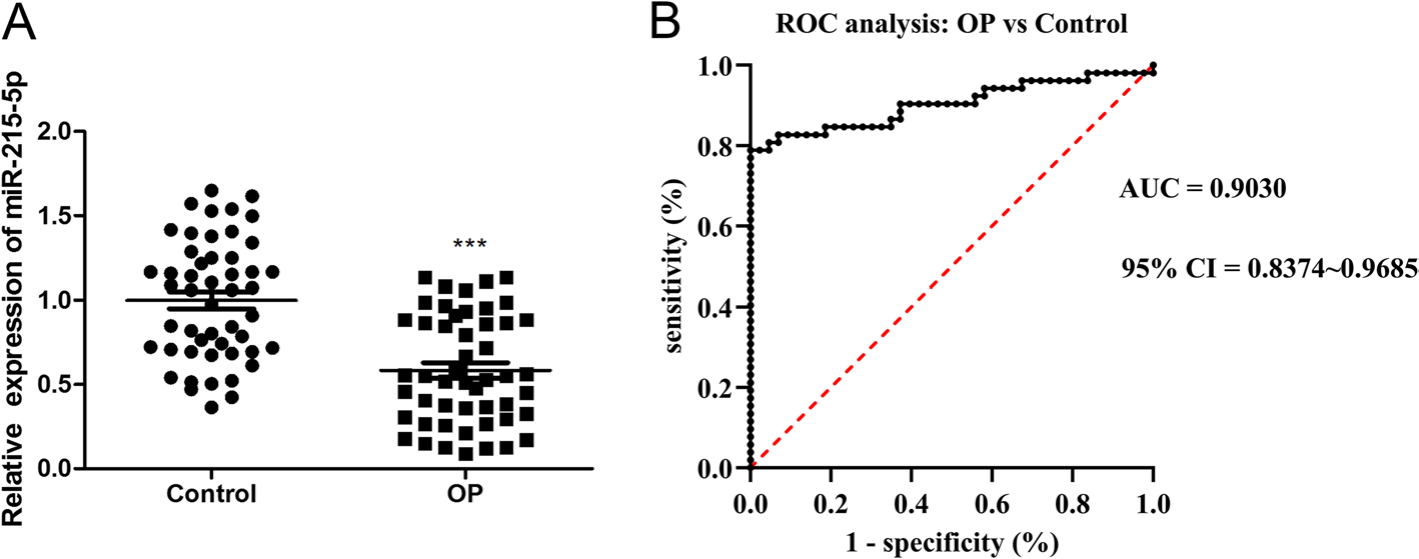
Aberrant expression of miR-215-5p in OP patients. **A** qRT-PCR analysis was performed to evaluate expressions of miR-215-5p in serum samples from OP patients and healthy controls. **B** Diagnostic value of miR-215-5p in discriminating OP patients from healthy controls

To confirm the significance of miR-215-5p in OP, we first collected samples from OP patients and healthy controls. By qRT-PCR analysis, we found that miR-215-5p was dramatically reduced in OP samples compared with healthy controls, suggesting its potential to protect OP development (Fig. 1A). Moreover, receptor functionality (ROC) analysis showed that miR-215-5p could discriminate OP patients from healthy controls with high specificity (100%) and high sensitivity (78.85%) and precision (threshold = 1.7885, AUC = 0.9030, 95% CI = 0.8374 ~ 0.9685; Fig. 1B). Finally, we divided OP patients into two groups based on the average of OP samples: a high expression group and a low expression group. The above results could deduce that miR-215-5p may play a role as a potential biomarker and be involved in the OP pathogenesis.

### miR-215-5p mediated hBMSCs osteogenic differentiation

**Fig. 2:**
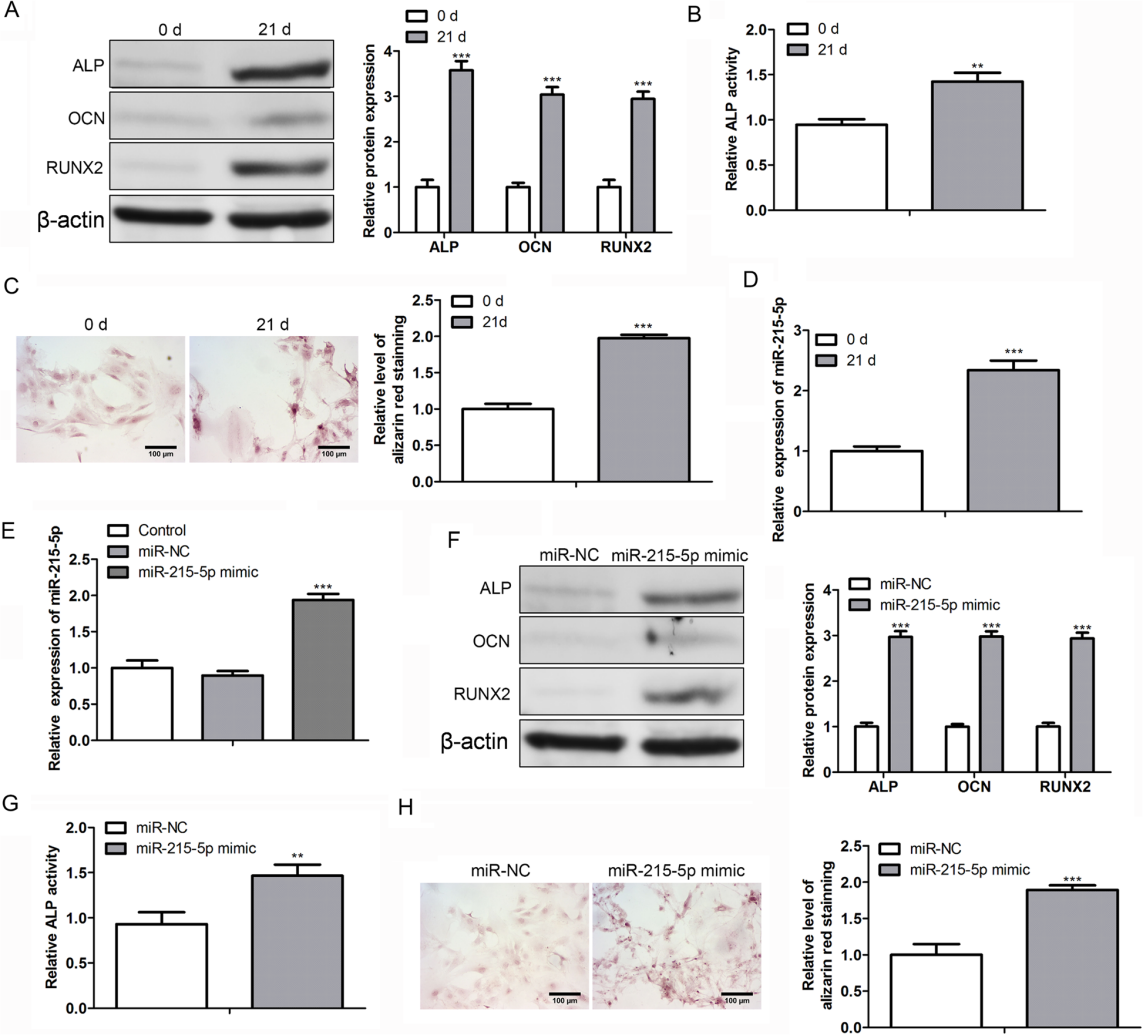
Impacts of miR-215-5p on hBMSCs osteogenic differentiation. **A** Western blot assay was conducted to detect ALP, OCN and RUNX2 expressions. **B** ALP activity was examined to evaluate the mineralization of hMBSCs. **C** Alizarin red staining was used to measure calcium deposits in hBMSCs. **D** Expression of miR-215-5p after β-glycerophosphate treatment in hBMSCs. **E** Transfection efficacy of miR-215-5p was verified by qRT-PCR analysis. **F** Effect of miR-215-5p transfection on ALP, OCN and RUNX2 was confirmed by western blot assay. **G** Effect of miR-215-5p on ALP activity in hBMSCs. **H** Effect of miR-215-5p on Alizarin red staining result in hBMSCs

First, we treated hBMSCs with β-glycerophosphate to mimic the osteogenic differentiation process. After 21 d of treatment, we found that osteoblast-related proteins ALP, OCN and RUNX2 levels were evidently increased as verified by western blot assay (Fig. 2A). Then, we detected ALP activity and calcium deposits in β-glycerophosphate-treated hBMSCs. As depicted in the Fig. 2B and C, the ALP activity and Alizarin red staining were significantly increased after treatment. The abovementioned results implied that β-glycerophosphate treatment successfully induced osteogenic differentiation.

From Fig. 2D, we found a significant augment in the miR-215-5p expression after the induction of β-glycerophosphate in hBMSCs, suggesting that miR-215-5p may be implicated in regulating the differentiation progress of osteoblasts. To further confirm the role of miR-215-5p in OP, we transfected hBMSCs with miR-215-5p or its negative control (miR-NC) without treatment with β-glycerol phosphate. Figure 2E confirmed the success of transfection, as the miR-215-5p level was overtly promoted in the mimetic group in comparison with the miR-NC group. Western blot assay then elaborated that miR-215-5p mimics could increase ALP, OCN, and Runx2 levels compared with the miR-NC group (Fig. 2F). In addition, ALP activity and alizarin red staining increased in the mimic group (Fig. 2G and H). Overall, these results demonstrated that the miR-215-5p simulator and the osteoblast inducer β-glycerophosphate have similar effects on the osteogenic differentiation of hBMSCs.

### miR-215-5p directly targeted XIAP

**Fig. 3:**
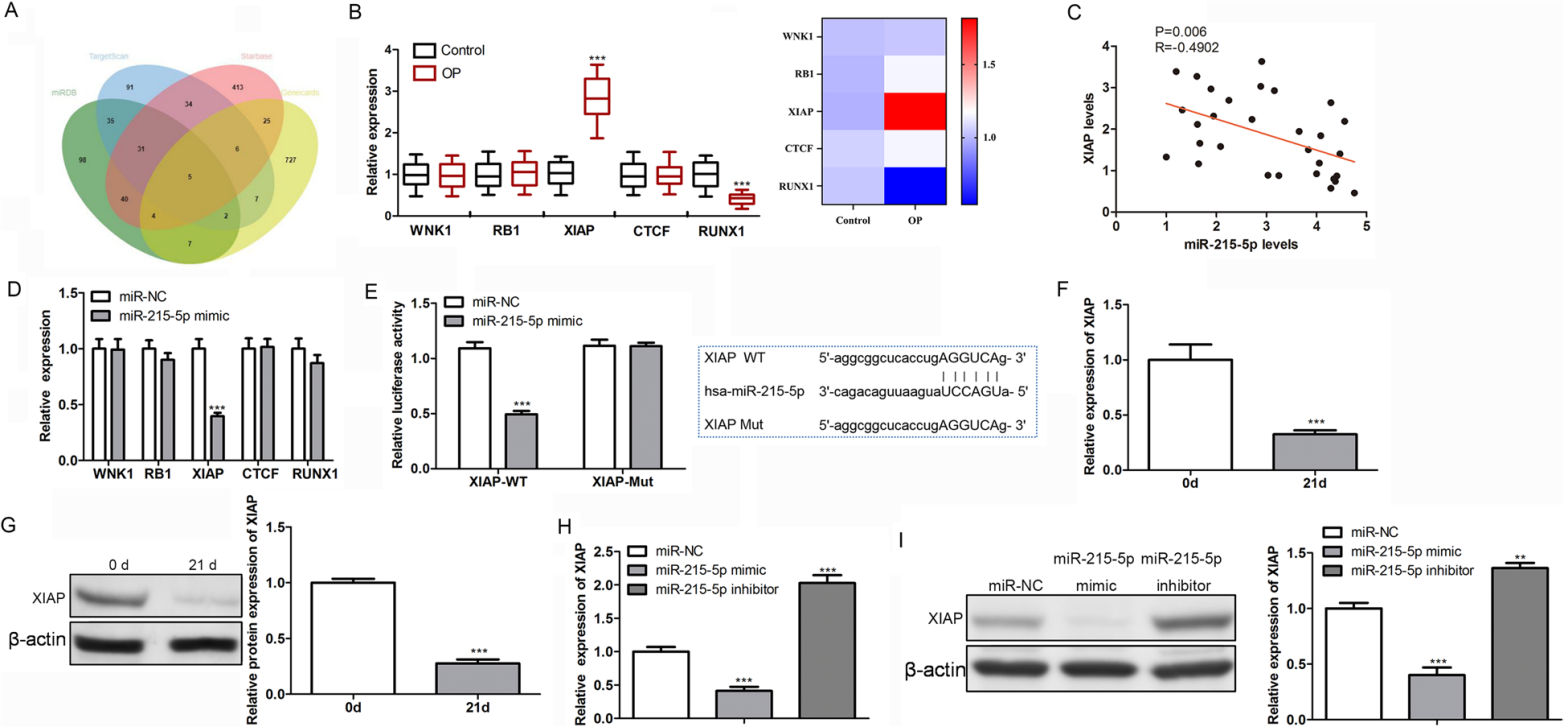
Targeted relationship between miR-215-5p and XIAP. **A** Potential targets of miR-215-5p were predicted by miRDB, TargetScan and Starbase. **B** Abnormal expressions of WNK1, RB1, XIAP, CTCF and RUNX1 in OP patients and healthy controls. **C** Correlation between miR-215-5p and XIAP in OP samples. **D** Expressions of WNK1, PB1, XIAP, CTCF and RUNX1 after transfection with miR-215-5p mimics in hBMSCs. **E** Dual-luciferase reporter assay confirmed the targeted relationship between miR-215-5p and XIAP. **F** qRT-PCR assay was conducted to examine the change of XIAP level before and after β-glycerophosphate treatment in hBMSCs. **G** Western blot assay was conducted to examine the change of XIAP level before and after β-glycerophosphate treatment in hBMSCs. **H** Effect of miR-215-5p transfection on XIAP level was determined by qRT-PCR analysis. **I** Effect of miR-215-5p transfection on XIAP level was determined by western blot analysis

Downstream targets for miR-215-5p are predicted using online tools mirdb, Targetscan and Starbase. The obtained target is then crossed with the OP-related target in GeneCards (GIFTS ≥ 45) to obtain the downstream target genes Wnk1, Rb1, XIAP, CTCF, and Runx1 (Fig. 3A). Moreover, qRT-PCR results showed no significant difference in WNK1, RB1, and CTCF levels between OP samples and healthy controls. Runx1 was significantly reduced, whereas XIAP was enhanced in OP samples (Fig. 3B). Moreover, the correlation analysis of Fig. 3C confirmed that miR-215-5p was inversely correlated with XIAP levels in OP samples. Therefore, the results from clinical samples demonstrate that only XIAP is suitable for downstream targets of miR-215-5p in OP.

Next, the relationship between miR-215-5p and XIAP was investigated by a series of functional experiments. We transfected hBMSCs with miR-215-5p or miR-NC mimics. The qRT-PCR analysis showed that only XIAP levels were significantly reduced in the miR-215-5p group (Fig. 3D). Dual-luciferase reporter assay then confirmed that the transfection of miR-215-5p mimic significantly reduced luciferase activity in the XIAP-WT group compared with the miR-NC and XIAP-WT transfection groups (Fig. 3E). No significant difference was found in the XIAP-MUT transfection group. In vitro, we observed a significant decrease in XIAP levels after 21 days of β-glycerophosphate treatment compared with the untreated group (Fig. 3F and G). Finally, transfection experiments revealed that miR-215-5p mimics can hinder the levels of XIAP, whereas miR-215-5p inhibitor enhanced XIAP level (Fig. 3H and I). Overall, XIAP played the role of a downstream target of miR-215-5p in OP development.

### miR-215-5p targeted XIAP to mediate osteogenic differentiation

**Fig. 4:**
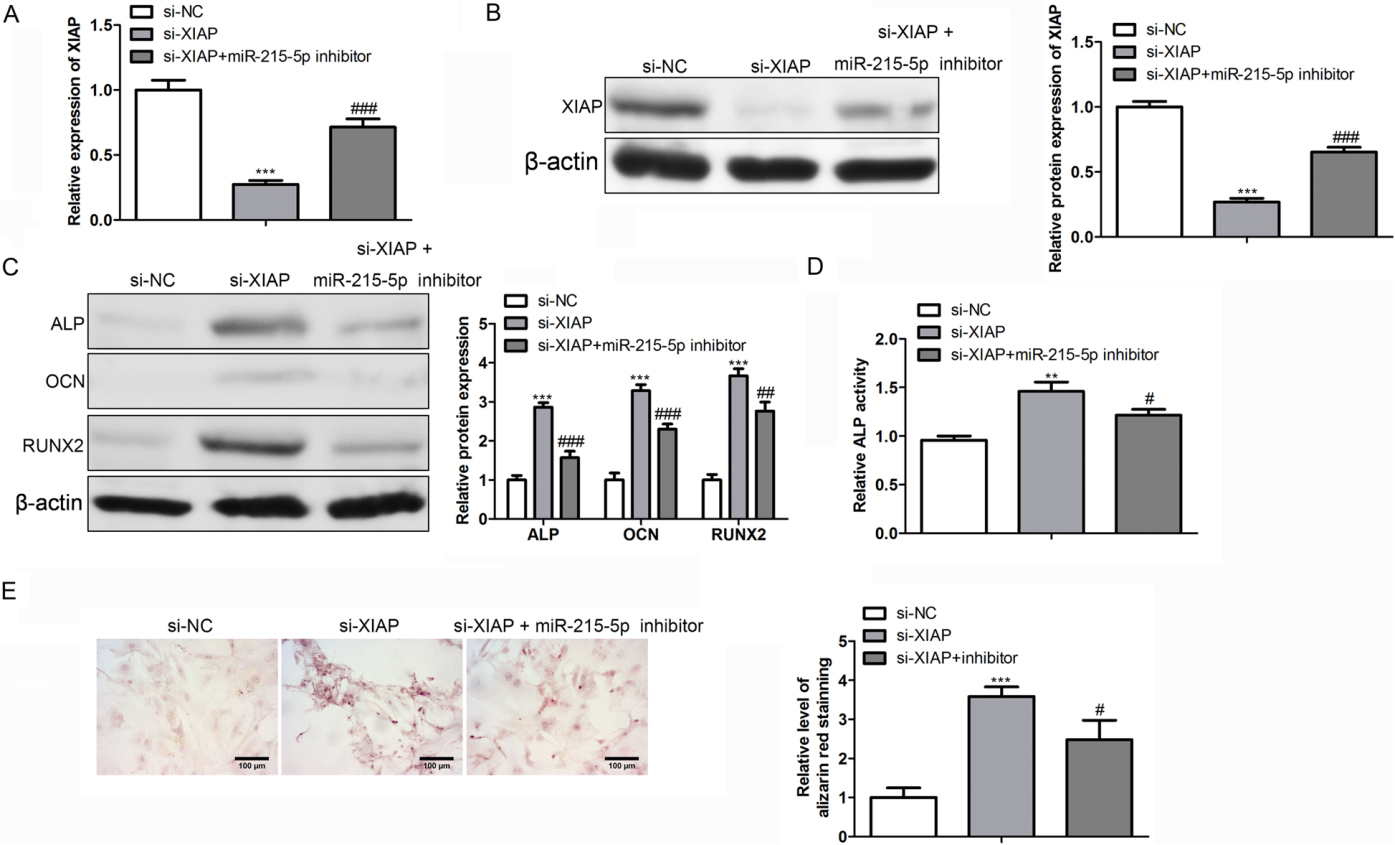
miR-215-5p mediated osteogenic differentiation via targeting XIAP. **A** Transfection efficacy of XIAP was determined by qRT-PCR analysis. **B** Transfection efficacy of XIAP was determined by western blot analysis. **C** miR-215-5p mediated ALP, OCN and RUNX2 levels via regulating XIAP. **D** ALP activity. **E** Alizarin red staining results

As to reveal the regulatory mechanism of the miR-215-5p/XIAP axis in OP, we knocked down XIAP expression by transfecting si-XIAP in hBMSCs. The results in Fig. 4A and B elucidated that XIAP level was remarkably reduced in si-XIAP group; however, the inhibition of XIAP level could be partially restored by co-transfection with miR-215-5p inhibitor. Then, the western blot assay suggested that silencing of XIAP could enhance protein expressions of osteoblast-related markers ALP, OCN and RUNX2; whereas, the facilitated effect induced by si-XIAP could be counterbalanced by miR-215-5p inhibitor in part (Fig. 4C). Finally, the ALP activity and Alizarin red staining accumulation were notably potentiated by XIAP knockdown; nevertheless, the enhancement induced by si-XIAP could be partially reversed by miR-215-5p inhibitor (Fig. 4D and E). In sum, down-regulation of XIAP promoted the osteogenic differentiation of hBMSCs; however, this promotive effect was counteracted by co-transfection of miR-215-5p inhibitor.

## Discussion

The human skeletal system is in the dynamic regulation of bone formation and bone resorption [23]. When this relationship is imbalanced, it leads to loss of bone mass, decreased bone stability, increased risk of bone fragility, and eventually leads to OP [24]. The osteoblast is a bone-forming cell differentiated from mesenchymal progenitors [25]. It can specifically secrete bone matrix, playing a pivotal role in bone formation and reconstruction [26]. In OP, the ability of hBMSCs osteogenic differentiation may participate in OP development. For instance, sesamin could protect against OP aggravation by promoting hBMSCs osteogenic differentiation [27]. Li et al. [28] illustrated that high expression of miR-149-3p presented a high tendency to differentiate hBMSCs into osteoblast, serving as a prospective marker for OP treatment. In our study, we stimulated hBMSCs with β-glycerophosphate and L-ascorbic acid to induce osteogenic differentiation, as described before [29]. The results demonstrated that ALP activity and Alizarin red staining were increased along with enhanced osteoblast marker expressions. Collectively, the data elaborated that β-glycerophosphate and L-ascorbic acid successfully induced hBMSCs differentiated into osteoblasts.

MiR-215-5p is located on Chr1: 220, 117, 853–220, 117, 962 (GrCH38/Hg38) and has a size of 110 bases. It regulates tumorigenesis, which has been widely reported. For example, the tumor-inhibitory effect of miR-215-5p has been demonstrated in colorectal cancer [30], breast cancer [31], and papillary thyroid carcinoma [32]. In the field of bone, Liu et al. [33] illustrated that miR-215-5p is a candidate against cancer in multiple myeloma. Another study by Monterde-Cruz et al. [34] validated that miR-215-5p from a Mexican population sample is upregulated in osteosarcoma and acts as a potential biomarker for the diagnosis of osteosarcoma. Another study found that miR-215-5p was markedly reduced in OP samples in contrast to non-OP or bone mass-reduced samples [21]. Consistent with previous studies, a remarkable decrease in miR-215-5p levels was observed in OP samples. Moreover, miR-215-5p served as a viable biomarker for OP diagnosis. In vitro, we found that treatment with β-glycerophosphate in hBMSCs significantly promoted the levels of miR-215-5p. At the same time, overexpression of miR-215-5p promoted the differentiation of osteoblasts from HBMSc. Collectively, miR-215-5p can promote the differentiation of osteoblasts and thus contribute to OP therapy.

As known, miRNAs are involved in mediating the development of various diseases by targeting the translation of mRNA target proteins. Previous studies have elucidated the targeted relationship between miR-215-5p and XIAP [35–37]. Consistent with these studies, functional experiments we examined found that XIAP was a downstream target of miR-215-5p and showed a negative correlation with miR-215-5p in OP. Moreover, XIAP was significantly increased in OP patients, which is consistent with previous conclusions [2, 38]. After stimulation of osteogenic differentiation, we observed a decrease in XIAP levels in HBMSCs. Functional gain and loss experiments suggest that XIAP silencing promotes osteogenic differentiation and the protective effect is partially reversed by miR-215-5p inhibitor.

In sum, miR-215-5p facilitated hBMSCs osteogenic differentiation by targeting XIAP, shedding new insights for OP therapeutic strategies.

## Supplementary Information

**Additional file 1. **

**Additional file 2. **

**Additional file 3. **

## Data Availability

All data generated or analysed during this study are included in this published article.
